# Pseudotail, scoliosis and syndactly, is it new syndrome?

**DOI:** 10.1186/1748-7161-10-S1-P1

**Published:** 2015-01-19

**Authors:** Mohammad D Alfawareh, Tamer Oreif, Anwar Elwan Mohammad

**Affiliations:** 1 King Fahd Medical City, Saudi Arabia; 2 Pronce Mohammad Bn Abdulaziz Hospital, Saudi Arabia

## Introduction

Pseudotail is uncommon congenital malformation of the spine, it contains bone, cartilage, notochord and elements of spinal cord. Usually presented as protrusion of the coccygeal vertebra.

It may come as solo deformity or in association of tethered cord, myelomeningoceol and other spine deformity, can be part of syndrome, never been reported.

It's different from true tail, which usually protrude outside the skin as true cutaneous appendages that protrude at the lumbosacral region.

## Purpose

We are presenting a case series; four patients; two sisters, their brother and one cousin, all presented with triad; pseudotail, congenital scoliosis and polysyndactaly in both hands and feet.

This can be syndrome we call it Alfawareh syndrome.

## Methods

We are reporting new syndrome, presented in a family, two sisters, their younger brother and their cousin.

All presented with the triad, polysyndactly was obvious at birth, hard mass bony mass at the tail bone, with pain, uncomfortable setting, in addition of complex polydactaly and scoliosis.

X ray was reviewed, CT scans and also MRI was reviewed for all patients.

Scoliosis correction was done in two out of four; pseudotail was excised in two out of four.

Pathology report also was reviewed.

## Summary

We are reporting a family of two sisters, their brother and cousin presented at different ages 4 to 14 years.

All presented with triad of polysyndactly, in all was managed and operated by hand surgeon at another institution, scoliosis and pseudotail.

Physical examination was unremarkable but syndactlay of hands and feet, rib hump and pseudotail, skin dimple, hard bony mass protruding posteriorly, tender on pressure and atrophic overlaying skin.

Two were underwent scoliosis surgery and pseudotail excision others on observation.

## Results

Successful results, both of them also get scoliosis surgery, the oldest get permanent fusion while the little brother gets growing rods.

The other two patients still on regular follow up.

## Conclusions

Pseudotail can be part of syndrome, we reported four patients- case series presented with triad of pseudotail, scoliosis and syndactlay, we name it Alfawareh Syndrome.

